# Thermography Sensor to Assess Motor and Sensitive Neuromuscular Sequels of Brain Damage

**DOI:** 10.3390/s24061723

**Published:** 2024-03-07

**Authors:** Alessio Cabizosu, Daniele Grotto, Alberto López López, Raúl Castañeda Vozmediano

**Affiliations:** 1 THERMHESC Group, Chair of Ribera Hospital de Molina San Antonio, Catholic University of Murcia (UCAM), 30107 Murcia, Spain; d.grotto3@gmail.com; 2 ASTRPACE, Association for the Treatment of People with Cerebral Palsy and Related Pathologies, 30007 Murcia, Spain; alberto.lopez@astrapacemurcia.es; 3 Faculty of Medicine, Universidad Francisco de Vitoria, 28935 Madrid, Spain; raul.castaneda@ufv.es

**Keywords:** thermography, brain damage, stroke, neuromuscular diseases

## Abstract

Introduction. The aim of this study was to observe the validity, diagnostic capacity, and reliability of the thermographic technique in the analysis of sensitive and motor sequelae in patients with chronic brain damage. Method. A longitudinal descriptive observational study was performed. Forty-five people with impairment in at least one anatomical region participated in and completed this study. All patients who had become infected by SARS-CoV-2 in the past year were excluded. Thermographic measurement was conducted, and the Modified Ashworth Scale and Pressure Pain Threshold was analyzed. Results. A high correlation between two times of thermography data was observed. The Spearman correlations obtained between the Ashworth score on each leg and the temperature given by thermography were all significant. Discussion and conclusions. Despite the above, the Spearman correlations obtained between the PPT in each leg and the temperature offered by thermography were not significant in any of the measurements. For this reason, thermography is a potential tool for the diagnosis and assessment of neuromuscular motor sequelae, but not for sensitive sequelae, after brain injury. Nevertheless, for the time being, no statistical relationship has been observed between the data reported by thermography and PPT; thus, future studies are needed to further investigate these results.

## 1. Introduction

DITI refers to a diagnostic and medical imaging tool that provides a digitized thermal pattern of a specific ROI [1]. Based on the capture of infrared radiation emitted by the skin’s surface, a thermal pattern is generated and converted into a digitized image. The physical process on which this technique is based is the analysis of the molecular agitation of matter, given that the greater the molecular agitation, the greater the heat released [2] and, consequently, the greater the infrared emission of the surface analyzed, thus making it possible to digitally observe physiological changes in different anatomical areas [3].

Because this technique provides data in relation to the skin’s surface, DITI has initially been mainly used in the analysis of skin-related diseases [4,5]. However, once it was demonstrated that thermographic asymmetries at the cutaneous level may represent situations of physiological imbalances at deeper levels, numerous studies have been carried out to evaluate physiological and metabolic responses in different diseases [6,7].

In the past decade, due to the numerous advances in the standardization of thermographic protocols and because thermography is a non-invasive, non-contact, and low-cost tool in relation to other diagnostic imaging techniques [8,9], the cutaneous thermal response has been the subject of numerous studies aimed at determining the pathological and functional states of patients based on dermal thermoregulatory processes [10,11]. In addition, it should be noted that this tool is very versatile and easily transportable, since its weight and size are reduced in relation to other conventional diagnostic techniques. This greatly facilitates its use in different clinics and laboratories, and is a very valid tool for monitoring metabolic responses in situ today, even in sports competitions [12].

Currently, in health sciences, there are several studies that analyze the role of DITI in different areas [13,14,15]. Likewise, in the neurological field, numerous studies have observed the validity of this technique in relation to muscular trophic processes, observing that there are structural and functional alterations in atrophied muscles that can be correlated to thermographic changes observed at the cutaneous level [16,17]. To show the correlation between the thermal characteristics of the body at the surface level and deep muscle activation in patients with neuromuscular disorders, thermographic tests have been associated with functional motor tests and other biomedical diagnostic images [18,19]. This is because, unlike MRI and axial tomography, clinical thermography provides more physiological than structural values. Nevertheless, the fact that it does not represent a health risk for patients with metallic or electronic implants, as is the case of MRI; does not provide ionizing radiation, as is the case of axial tomography; and has the capacity to analyze large anatomical regions in a short time, unlike ultrasound, has projected this technique in the area of neuromuscular diseases as a very efficient and novel tool. In studies related to the rehabilitation of neurological damage evaluated by clinical thermography, changes in joint function have been related to the microcirculation of spastic extremities. According to these studies, there is a direct relationship between cutaneous microcirculation and the degree of functional impairment because, after the therapeutic protocol, an increase in cutaneous thermal symmetry between extremities was observed, accompanied by a functional motor improvement [17,20].

These studies suggest that thermography could be a valid tool in the assessment and monitoring of the level of muscle atrophy in relation to motor function. Since, in these types of patients, the decrease in metabolic and functional activity of the muscular system seems to be directly related to changes in the skin’s surface temperature, the analysis of the ΔT by DITI could be transcendental for understanding the physiology and functional response of trophic pathologies associated with permanent functional dependence [16,17,20]. If this were to be demonstrated in future studies, it would make it possible, on the one hand, to speed up patient evaluation processes and times compared to other diagnostic techniques, and on the other hand, to allow professionals to perform this evaluation without contact or ionizing radiation. Finally, the evaluation and follow-up of patients could be objectified and quantified by means of objective quantitative values and not subjective qualitative values, which was the main scale of evaluations used until now.

Currently, due to the novelty of the topic, there is not enough information to generate a consensus on DITI responses in relation to sensory and motor alterations in patients with brain damage or neurodegenerative diseases; however, the functional and structural sequelae associated with damage to the nervous system represent an important challenge for researchers and health personnel due to the large number of new cases that occur annually and the associated disabilities [21,22].

To clarify these possible relationships, the aim of this study was to observe the validity, diagnostic capacity, and reliability of the thermographic technique in the analysis of sensitive and motor sequelae in patients with chronic brain damage.

## 2. Methodology

### 2.1. Study Design

A longitudinal descriptive observational study was performed. The study was approved by the Ethics Committee of the Catholic University of San Antonio of Murcia (CE102107). Written informed consent from the patient or the consent of their relatives was obtained. All patients who had suffered brain damage with more than 5 years of evolution, and who had shown motor or sensitive impairment in at least one anatomical region according to the neurological reports obtained, were included. All patients who had become infected by SARS-CoV-2 in the past year were excluded, as its potential influence on the thermography response is not known. Also, all patients who did not comply with the TISEM check-list recommendations [23], as outlined in the information sheet, as well as those who could not understand the commands given throughout the tests, were excluded. A specialist in neuromuscular damage classified patients according to age, sex, BMI, and functional diagnosis.

### 2.2. Participants

No limits on age, sex, or race were established. Twenty-four Spanish men and twenty-one Spanish women participated in and completed this study (age: mean ± SD = 33.8 ± 7.4; IMC: mean ± SD = 26.5 ± 5.3). Fourteen patients with PMI, fourteen patients with SD, seven patients with RH, three patients LH, and seven patients with TE were included. This work was carried out using an expanded sample of a previous preliminary study [24]. The patients’ characteristics are presented in Table 1.

### 2.3. Procedures

A blinded researcher specializing in the assessment of neuromuscular damage measured muscular impairment according to the modified Ashworth Scale to evaluate the patients’ restriction and resistance to bending of the ankle [25]. The sensitivity of pain in gastrocnemius ROI was measured via PPT, as described by other authors [26,27], while 2 researchers recorded thermal patterns. For the thermographic measurement, the recommendations of the TISEM protocol [23] were followed. The algometer used was the Wagner FORCE TENTM FDX model (Greenwich, CT, USA). This model is intended for hand-held force testing, with a pistol grip handle operating on a rechargeable battery for portable use, and has a margin of error of ±0.3%.

### 2.4. Familiarization and Clinical Exam

The clinical examination included an analysis of the medical records by a neurologist specializing in brain damage and of basal temperature through thermometry to rule out febrile states and thus confirm that the volunteer was fit to participate in the study. In the familiarization session, participants were placed in the algometric and thermographic evaluation position to familiarize them with the stimuli and distances.

### 2.5. Thermography

The thermograph used was the Flir E75 model (Wilsonville, OR, USA). This thermograph has a thermal sensitivity of <0.04 °C and registers from −20 °C to +120 °C. The emissivity was established as 0.98, as suggested by other studies [28]. For thermalization, for 15/20 min, all participants remained in a room with an area of 15 m^2^, at a temperature between 20 °C and 23 °C, a humidity level of 40% (±0.8%), and an atmospheric pressure of 1 ATM. Body temperature was checked with a digital thermometer to rule out alterations in base temperature. To guarantee a depth analysis of the images, the ROIs were marked on the digitized photo and the thermographic image was subsequently selected. Two blinded researchers with more than 5 years of experience in thermography recorded images of the posterior legs on different days and times, with 1 week between measurements. As in previous studies, ROIs were identified from the lower edge of the popliteal fossa to 15 cm above the lower edge of the heel, thus allowing the gastrocnemius region to be analyzed [29]. Image processing was performed using the Flir IR research program/software [30]. Due to camera errors, capture errors, non-flat body shape, and the variability of the left and right ROI definitions, if the ΔT was less than or equal to 0 to 0.5, it was considered to have high reliability. If it was in the range between 0.5 and 1, it was considered reliable; if it was in the range between 1 and 1.5, it was considered to have low reliability; and if it was greater than 1.5, it was considered unreliable, as already described by other authors [31,32].

### 2.6. Statistical Analysis

To assess thermography as a diagnostic tool and the criterion validity of the instrument, Student’s *t*-test was applied for leg measurements in which the temperature was normally distributed according to the Shapiro–Wilk test (*p* > 0.05) and the Wilcoxon test for those where the null hypothesis of normality was rejected (*p* < 0.05). To assess the convergence validity between the temperature and the modified Ashworth Scale and Temperature and PPT, Spearman correlations were used based on the results in each of the Shapiro–Wilk normality tests (*p* < 0.05), and graphs were produced for visualization. Possible confounding effects of variables such as age, sex, or IMC were controlled to rule out a possible spurious relationship between Ashworth and thermography. The critical level obtained for each of them was studied assuming a level of significance α = 0.05 (a Type I error of 0.01 or similar was not considered, as the sample was small and this would have further increased the Type II or Beta error). Also, the magnitude of the effect was assessed, according to conventional criteria [33], as small (0.10–0.30), moderate (0.30–0.50), or large (>0.50). To evaluate the reliability of thermography as a consistent diagnostic tool at both time points, ICC was estimated using a two-way random effect model and interpretated as described by another author [34].

## 3. Results

### 3.1. Concurrent Validity of Thermography, Diagnostic Capacity, and Reliability of Thermography

#### 3.1.1. Significant and Descriptive Inferiority of all Temperatures with Respect to 32 °C

The Shapiro–Wilk tests reported normality in the distribution for temperatures referring to LL 1° (*p* = 0.119) and LL 2° (*p* = 0.241), unlike RL 1° (*p* = 0.014) and RL 2° (*p* = 0.006) (excluding LH and RH patients, respectively). As can be seen in Table 2, of the four temperature measurements taken, the two taken on the RL (excluding LH patients) obtained maximum values lower than 32° (RL 1° = 31.4; RL 2° = 31.5), as well as the first measurement on the LL (excluding RH patients) (LL 1° = 31.6). Although the second measurement made on the LL obtained a maximum temperature slightly higher than 32° (LL 2° = 32.3), 75% of the sample found values lower than 29.8°. In addition, each of the four measurements was statistically rejected as having a mean value greater than or equal to 32° (*p* < 0.001).

#### 3.1.2. Differences between Times of Temperature Differences between Legs

Descriptively, it was observed that 86.66% of the sample (39 patients) registered differences between legs that did not vary by ±0.5 degrees between one time point and another, meeting the criterion established as “high reliability of the test”. As indicated in Table 2, those who failed to comply with the high reliability range of the test (differences of 0–0.5° between legs between two time points) were a total of six cases, which represents only 13.33% (statistically non-significant according to the above analyses). A total of 8.88% (four cases) registered differences between 0.5° and 1° (“reliable”); 2.22% (two cases) registered differences between 1° and 1.5° (“unreliable”); and another 2.22% (two cases) registered differences greater than 1.5° (“unreliable”). In fact, despite the differences found between the temporary measurements, the temperatures of each leg at the first time point correlated highly with the measurements made at the second (RL 1–2°: rho = 0.7471; LL 1–2°: rho = 0.6992), which shows that the differences resulting from measurements at different time points were highly consistent for the sample as a whole.

**Figure 1:**
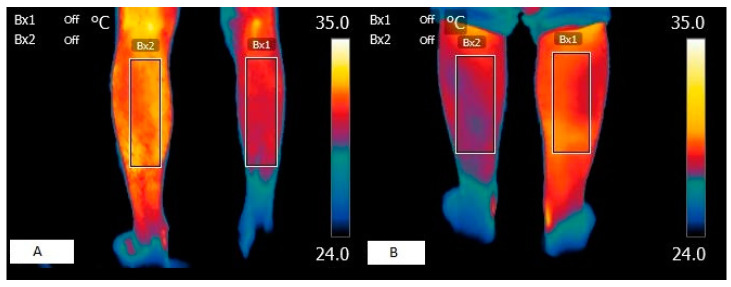
Thermographic images of a patients with muscular spasticity. (**A**) Skin surface image of patient with right hemiparesis. (**B**) Skin surface image of patient with left hemiparesis.

**Figure 2:**
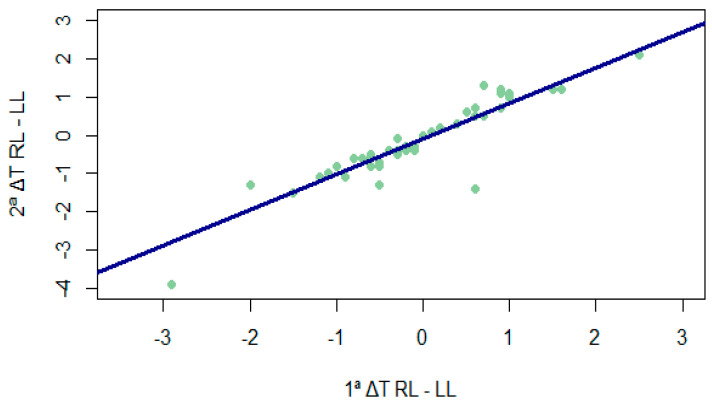
Correlation between temperature differences between legs at different times. 1ª ΔT RL-LL = temperature difference between the legs in the first measurement; 2ª ΔT RL-LL = temperature difference between the legs in the second measurement.

Although the difference in temperature between legs was significantly higher than 0.5° at both times (Figure 1) (1. ΔT RL-LL: V = 599.5, *p* = 0.014; 2. ΔT RL-LL: V = 646.5, *p* < 0.001), the differences between legs at the two time points were not equal to or higher than 0.5° (V = 118, *p* = 1), representing a high correlation (rho = 0.910; *p* < 0.001) between them (as can be seen in Figure 2).

#### 3.1.3. Temperature Differences between Times for Each Leg

Despite the differences found between the temporary measurements (Table 1), the temperatures of each leg at the first time point correlated highly with the measurements made at the second (PD 1–2°: rho = 0.7471, ICC = 0.635 [95%IC = 0.355–0.798]; PI 1–2°: rho = 0.6992; ICC = 0.603 [95%IC = 0.337–0.771]), which shows that the differences resulting from the measurements at different time points were highly consistent for the sample as a whole.

### 3.2. Convergent Validity with Other Instruments

#### 3.2.1. Relationship between Temperature and Ashworth Scale

**Figure 3:**
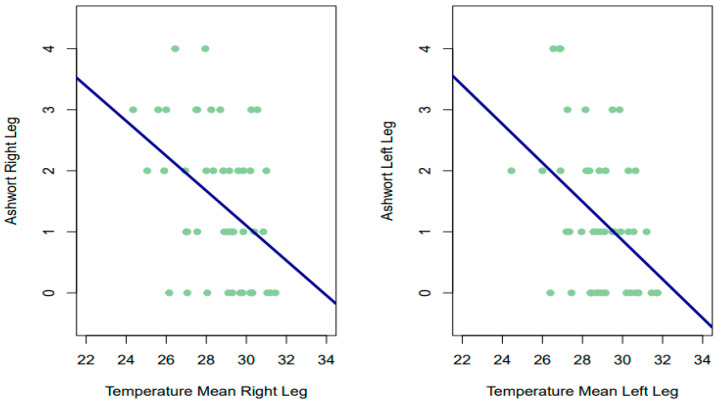
Relationship between temperature and Ashworth scale.

The Spearman correlations obtained between the Ashworth score on each leg and the temperature given by thermography (for the two time measures, as well as their mean) were all significant, with negative and moderate signs for both the right leg (RL 1°: rho = −0.381, *p* = 0.0097; RL 2nd: rho = −0.397, *p* = 0.00695; mean 1–2° RL: rho = −0.410, *p* = 0.0051) and the left leg (LL 1°: rho = −0.341, *p* = 0.02244; LL 2nd: rho −0.368, *p* = 0.01241; mean 1–2° LL: rho = −0.391, *p* = 0.0078). High temperature scores were observed to be associated with low scores on the Ashworth scale, and vice versa (Figure 3).

As possible confounding variables, age did not correlate significantly with the Ashworth scale for either leg (RL: rho = 0.008, *p* = 0.957; LL: rho = 0.0288, *p* = 0.851), nor did IMC (RL: rho = −0.113, *p* = 0.456; LL: rho = 0.0113, *p* = 0.941). There were also no significant differences in Ashworth scores between men and women on the right leg (*p* = 0.319) or on the left leg (*p* = 0.189).

#### 3.2.2. Relationship between Temperature and PPT

**Figure 4:**
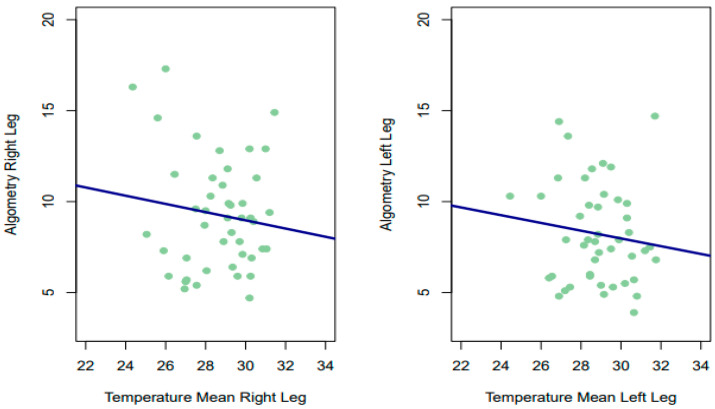
Relationship between temperature and algometry.

Despite the above, the Spearman correlations obtained between the PPT in each leg and the temperature offered by thermography (for the two time points, as well as their mean) were not significant in any of the measurements, being of small magnitude and slightly negative for almost all estimates for both the left leg (LL 1°: rho = −0.124, *p* = 0.415; LL 2nd: rho −0.158, *p* = 0.299; mean 1–2 LL: rho = −0.144, *p* = 0.344) and the right leg (RL 1st: rho = −0.0595, *p* = 0.6978; RL 2: rho = 0.0064, *p* = 0.9662; mean 1–2 RL: rho = −0.005, *p* = 0.975). Therefore, it has not been statistically rejected that the correlations obtained were different from 0. In the lower part of Figure 4, for example, we can see that patients with low scores on the algometry scale had low and high temperatures.

## 4. Discussion

The main objectives of this study were to observe the validity, diagnostic capacity, and reliability of the thermographic technique in the analysis of sensitive and motor sequelae in patients with chronic brain damage.

### 4.1. Thermographic Validity, Diagnostic Capacity, and Reliability

The main findings of this study show that each of the four measurements was statistically rejected as having mean values greater than or equal to 32° (*p* < 0.001). Although the ΔT between legs was significantly higher than 0.5° at both time points (T1: *p* = 0.014; T2: *p* < 0.001), the differences between legs at different time points were not equal to or higher than 0.5° (*p* = 1). Thus, we found a high correlation (rho = 0.910) between them. In fact, despite the differences found between the temporary measurements, the temperatures of each leg at the first time point correlated highly with the measurements made at the second (RL 1–2°: rho = 0.7471; LL 1–2°: rho = 0.6992), which shows that the differences resulting from the measurements at different time points were highly consistent for the sample as a whole.

Previous studies have suggested a temperature of 32° as a thermal reference value for the posterior region of the leg in healthy people [35,36]. Our results show that the mean temperatures of our patients were always lower than these values, which could be evidence of pathological conditions of the muscle structures analyzed. These results support previous investigations carried out in patients with the same clinical characteristics, where the mean temperature values of the posterior region of the leg were always statistically lower than 32 °C [24] or even 30 °C [37]. However, other authors have proposed reference values lower than 32 °C in the same ROI [29,38,39]; however, the thermal values provided by these authors, i.e., 30.7 °C [38], 30.2 °C [29], and 30.7 °C [39], are still higher than the mean values obtained in this work. Thus, even using lower reference values, the thermographic results obtained in the affected patients would show non-conventional temperatures given that, in our study, the patients presented a mean temperature lower than 29.5 °C in both legs at both times, like the results of similar works by other authors [37].

The difference between our data and the data proposed by the other authors may depend on the type of thermograph used; the time and place of measurement; and the ages, sexes, IMC, and profiles of the participants, since in most cases, they were athletes, which could be related to different metabolic activity compared to our sample. Nevertheless, due to the lack of homogeneity of information regarding normal temperature values in this anatomical segment, further validity studies are needed in order to definitively confirm this reference value in healthy individuals. Until then, it may be advisable to use the temperature differences between extremities, as suggested by other authors.

Regarding the results obtained on the ΔT on the same side and between sides at different times, our results support the findings previously described by other authors [24], because our results also show a statistically significant difference in the same leg, but not between legs at different times. In our study, in 86.6% of the cases, it was not possible to observe a temperature difference between both extremities that varied between the two time points by more than 0–5 °C. It is known from other authors that, depending on the time of measurement, different thermographic patterns can be generated in the same patient in the same ROI due to physiological and metabolic changes that are generated throughout the day or on different days [40,41]. For this purpose, other authors have suggested analyzing the ΔT between sides at different times in order to reduce biases in the results [42]. On the basis of these considerations and as proposed by other authors [17,43], it seems justified to think of a thermal model in which the ΔT observed in the same leg at different times is traceable to short-term temporal physiological processes, while the difference between legs at different times is traceable to the skin response and dependent on the amount of muscle trophic tissue, since this condition is not subject to short-term temporal variations. In this sense, future research could further clarify this model in order to understand the cutaneous physiological response in relation to muscular trophic processes.

### 4.2. Thermography Validity in Relation to Motor Ability

The main results obtained regarding the correlations between the modified Ashworth scale and the thermographic response showed that all correlations were significant, negatively, moderately, and bilaterally. It is known from other authors that the modified Ashworth scale is a reliable tool to assess motor sequelae after neurological damage, in fact, in studies performed with MRI, a good correlation between this scale and diagnostic results has been observed [44,45].

In the thermographic field, some authors have observed that, in patients with spastic hemiparesis, hypothermia coincides with a higher degree of motor impairment [17] as well as in our work (Figure 1). In patients with bilateral involvement, it has been found to be statistically significant only on one side and at a single moment [24]. It should be noted that the results obtained in the latter study may depend on the lack of homogeneity of the sample in terms of functional diagnosis and the small number of patients. In fact, in this study, which presents a larger sample, there was always a statistical correlation on both sides, showing that as the skin temperature decreases, greater motor impairment is observed. In complete or very severe peripheral nerve lesions, there is a decrease in temperature at the dermal level of the cutaneous tissue [46,47], since the perfusion of cutaneous tissues depends on muscle capillary density, and in denervated muscles, capillarity is lost and hypothermic patterns are observed [48]. In this sense, it is plausible to think that, in patients with sequelae of brain damage, such as atrophy or spasticity, the metabolic processes of the deep thermoregulatory element are affected. This may be due, on the one hand, to the low kinetic activity of the myocytes, and on the other hand, to the decrease in motor units due to the substitution of healthy muscle fibers for intramuscular fat infiltration [49]. However, other authors [50] have observed that despite the affected motor capacity in patients with central neurological lesions, the skin temperature of the masticatory muscles does not undergo significant changes, suggesting that further studies may better clarify these processes. Nevertheless, it should be noted that the small number of patients recruited in this study [50] could be an element of bias, as in studies with patients with the same clinical characteristics in the lower extremities, significant differences between the healthy side and the affected side have been observed via thermography, describing lower temperatures on the affected side compared to the healthy side [37].

Our results lead us to think that the mechanisms of supraspinal reorganization, in maintaining the balance of functions, could be at the basis of the thermal challenges observed. It is known that ischemic brain damage generates central neuroinflammatory responses aimed at preparing the brain for repair by means of future plastic processes. However, depending on the intensity of the neuroinflammatory process, intracranial rewarming processes can be observed that can generate damage at the level of the hematoencephalic barrier, hindering neurogenesis and angiogenesis and generating worse results in neurological, motor, and sensory functions [51,52]. After suffering brain damage, due to the death of neuronal pathways, the injured cortical areas stop “recognising” or “feeling” the anatomical region of reference in an optimal and efficient manner, generating functional sequelae in terms of motor and sensitive action [53,54]. This is reflected in consequences such as rigidity, spasticity, atrophy, sarcopenia, and loss of sensitivity. These conditions could affect the capillarity of muscle tissue due to the increase of avascular adipose tissue at muscle level versus vascularized healthy fibers [55], resulting in abnormal infrared patterns and muscular stiffness. In this sense, we consider the results of this study to be of great interest, because, if they could be replicated in future studies, they could form a basis for the evaluation and follow-up of pharmacological or therapeutic interventions due to trophic or degenerative diseases in a fast, non-invasive, and objective manner.

### 4.3. Thermography Validity in Relation to Sensitive Ability

The main results obtained regarding the correlations between the sensitive ability and the thermographic response showed that no correlations were significant, although they were slightly negative. It is known that the PTT pain measurement test can be used as an instrument for the assessment of hypoalgesia or high pain tolerance, as well as hyperalgesia or low pain tolerance [56]. Some authors have observed, with clinical thermography and PTT, that acute inflammatory processes also increase pain sensitivity as the temperature increases [57,58], while in chronic pathologies, no correlation has been observed between the decrease in temperature and the decrease in pain sensitivity [59]. In patients with brain lesions, pain sensitivity is correlated to somatosensory abnormalities of the hypoalgesia type [60], in addition to thermal and sensory differences according to the level of spastic involvement. This author observed differences according to age, with older patients showing greater sensitivity to pain compared to younger patients, and the affected sides of elderly patients showing greater sensitivity than those of younger patients [60] In this regard, our results contrast with the data provided by these authors, since this clinical situation regarding thermal differences and PTT results was not observed. This discrepancy in performance may be due to the degree of homogeneity of the lesion, which was much higher as all participants were hemiplegic patients, whereas in our study, we included several types of neurological sequelae. We believe that the sample size of the study and the heterogeneity of brain lesions could have diluted potentially significant findings. Increasing the sample size and stratifying participants based on the type and severity of their brain injury could improve the sensitivity of thermography to detect subtle changes associated with PPT. It is certainly of great interest to determine whether lower scores in temperature are accompanied by higher scores in PPT and lower scores in motor functionality, even in the presence of diseases with central sensitization, something that has been proposed by many authors and that contrasts with more recent evidence. To further clarify this situation, we believe that it would be interesting to conduct a study in patients with only one affected hemibody to observe whether there is a statistical correlation, on both the affected and healthy sides, regarding these three variables. This would not only help to clarify the relationship between cutaneous thermal processes and neurological sequelae, but could also open new lines of research in which to perform experimental studies with different therapeutic or diagnostic techniques using the cutaneous response as a biomarker. Combining thermography with other diagnostic tools, such as MRI or electromyography, could offer a more comprehensive assessment of neuromuscular function and sensitivity. This multimodal approach might uncover correlations which are not evident when thermography is used in isolation.

According to the results obtained in this study, thermography could be a useful tool to determine the level of motor, but not sensitive, involvement in patients with musculoskeletal sequelae secondary to brain damage. The small sample and the low homogeneity of the sample in this study represent a limiting factor. Future studies on the activation of the musculoskeletal and sensitive systems are needed, as this could influence the motor response, which in turn may affect the optimization of therapeutic protocols. This would help to understand how different levels of functional activation in this population could result in different skin surface temperature responses, and how they could help in the assessment and monitoring of the neurological patients in motor and, perhaps, sensitive analysis. We believe that the small sample size may have reduced the study’s statistical power, although we are confident that the results obtained in this study may help to develop future research.

## 5. Conclusions

Our results support the possibility of using thermography as a diagnostic and assessment tool for neuromuscular motor sequelae, but not for sensory sequelae, after brain injury. They show a direct relationship between the thermography results and the modified Ashworth scale, but not between thermography and PPT. Because, for the moment, no statistical relationship has been observed between the data reported by thermography and PPT, future studies are needed in order to deepen these results.

## 6. Limitation of the Study

An important limitation of this study is the small number of participants and the lack of similar studies for comparison of the results obtained. The possible impact of the small sample size on the generalizability of the study and the increase in type I errors in each of the hypothesis tests performed should be considered. Future studies with larger sample sizes would increase the power of the analyses and strengthen the validity of the results. However, it should be noted that our study is currently presented as one of the studies with the largest number of samples, and these results may be of great use for future research. Another limitation of the study could be the use of sere E equipment, since these are sensors designed for electrical engineering and not for research in humans. Furthermore, despite having been validated in previous studies as a highly reliable diagnostic tool in humans, to improve their accuracy and reliability, these devices should receive an updated registration from the Food and Drug Administration (FDA) or the European Medicines Agency (EMA) to be classified as medical devices for research.

## 7. Future Direction

Based on the results found in our study, future research could evaluate how lower skin level temperatures in patients with brain damage affect motor and sensitive function in order to clarify these findings. It should be noted that the timing of thermographic assessments with the onset of brain injury and subsequent neuromuscular changes may significantly impact the findings. Conducting longitudinal studies could provide insights into the temporal dynamics of thermographic changes and their correlation with PPT and motor disease. Moreover, identifying and focusing on specific neuromuscular pathways or regions of interest that are most likely to show changes detectable by thermography could enhance the study’s ability to detect significant correlations with PPT and confirm motor disease. A future study on more superficial structures such as the patellar tendon or the Achilles tendon could further clarify the role of thermography in relation to motor and sensory alterations in this type of patient. This could help health professionals who want to investigate new treatments or follow-up techniques, as well as health professionals who want to evaluate the long-term evolution of a patient.

## Figures and Tables

**Table 1:** General baseline characteristics.

Characteristics	Total (N = 45)
Sex	
Woman	24 (53.4%)
Man	21 (46.6%)
Age	
Mean ± SD	33.8 ± 7.4
IMC	
Mean ± SD	26.5 ± 5.3
Right MAS	
Mean ± SD	1.4 ± 1.2
Left MAS	
Mean ± SD	1.2 ± 1.2
Right PPT	
Mean ± SD	9.3 ± 3.1
Left PPT	
Mean ± SD	8.2 ± 2.7

**Table 2:** Reference thermography value for the patients affected in each leg.

	RL1°	LL 1°	RL 2°	LL 2°
N = 45	N = 42 ^a^	N = 38 ^b^	N = 42 ^a^	N = 38 ^b^
Mean ± SD	29.1 ± 1.8	29.1 ± 1.5	28.2 ± 2.2	28.3 ± 2.1
Median IQR [25–75%]	29.4 [28.3–30.4]	29.1 [28.0–30.4]	28.8 [26.0–29.8]	28.7 [26.3–29.8]
Range	23.9, 31.4	24.5, 31.6	23.9, 31.5	24.3, 32.3
*p* value	<0.001 *	<0.001 **	<0.001 *	<0.001 **

RL: right leg; LL: left leg; ^a^ = not including the 3 LH patients; ^b^ = not including the 7 RH patients. * Wilcoxon test, H_0_: *Mdn*_Y_ ≥ 32°; H_1_: *Mdn*_Y_ < 32°; ** Student’s *t* test, H_0_: μ_Y_ ≥ 32°; H_1_: μ_Y_ < 32°.

## Data Availability

Data generated or analyzed during this study are included in this article.

## Abbreviations

ATM	Atmosphere
BMI	Body mass index
DITI	Digital infrared thermal imaging
ΔT	Difference of temperature
HYP	Hypotonia
ICC	Interclass correlation coefficient
LH	Left hemiplegia
LL	Left leg
MAS	Modified Ashworth scale
MRI	Magnetic resonance Imaging
PMI	Psycomotor impairment
PPT	Pressure pain threshold
RL	Right leg
RH	Right hemiplegia
ROI	Region of interest
SD	Spastic diplegia
TE	Tetraplegia
T1	Time 1 of measurement
T2	Time 2 of measurement
TISEM	Thermographic imaging in sports and exercise medicine

## References

[1] Ring E.F.J., Ammer K. (2012). Infrared Thermal Imaging in Medicine. Physiol. Meas..

[2] Wu T., Luo Y., Wei L. (2017). Mid-Infrared Sensing of Molecular Vibrational Modes with Tunable Graphene Plasmons. Opt. Lett..

[3] Ring E.F. (1990). Quantitative Thermal Imaging. Clin. Phys. Physiol. Meas..

[4] Amson H., Vacheron C.-H., Thiolliere F., Piriou V., Magnin M., Allaouchiche B. (2020). Core-to-Skin Temperature Gradient Measured by Thermography Predicts Day-8 Mortality in Septic Shock: A Prospective Observational Study. J. Crit. Care.

[5] Magalhaes C., Vardasca R., Mendes J. (2018). Recent Use of Medical Infrared Thermography in Skin Neoplasms. Skin Res. Technol..

[6] Wilkinson J.D., Leggett S.A., Marjanovic E.J., Moore T.L., Allen J., Anderson M.E., Britton J., Buch M.H., Del Galdo F., Denton C.P. (2018). A Multicenter Study of the Validity and Reliability of Responses to Hand Cold Challenge as Measured by Laser Speckle Contrast Imaging and Thermography: Outcome Measures for Systemic Sclerosis-Related Raynaud’s Phenomenon. Arthritis Rheumatol..

[7] Fitzgerald A., Berentson-Shaw J. (2012). Thermography as a Screening and Diagnostic Tool: A Systematic Review. N. Z. Med. J..

[8] Ring E.F.J. (2006). The Historical Development of Thermometry and Thermal Imaging in Medicine. J. Med. Eng. Technol..

[9] Tattersall G.J. (2016). Infrared Thermography: A Non-Invasive Window into Thermal Physiology. Comp. Biochem. Physiol. A Mol. Integr. Physiol..

[10] Tansey E.A., Johnson C.D. (2015). Recent Advances in Thermoregulation. Adv. Physiol. Educ..

[11] Rustemeyer J., Radtke J., Bremerich A. (2007). Thermography and Thermoregulation of the Face. Head Face Med..

[12] Rodriguez-Sanz D., Losa-Iglesias M.E., Becerro-de-Bengoa-Vallejo R., Dorgham H.A.A., Benito-de-Pedro M., San-Antolín M., Mazoteras-Pardo V., Calvo-Lobo C. (2019). Thermography Related to Electromyography in Runners with Functional Equinus Condition after Running. Phys. Ther. Sport.

[13] Anzengruber F., Alotaibi F., Kaufmann L.S., Ghosh A., Oswald M.R., Maul J.-T., Meier B., French L.E., Bonmarin M., Navarini A.A. (2019). Thermography: High Sensitivity and Specificity Diagnosing Contact Dermatitis in Patch Testing. Allergol. Int..

[14] Modrzejewska A., Cieszyński Ł., Zaborski D., Parafiniuk M. (2021). Thermography in Clinical Ophthalmic Oncology. Arq. Bras. Oftalmol..

[15] Tan Y.K., Hong C., Li H., Allen J.C., Thumboo J. (2020). Thermography in Rheumatoid Arthritis: A Comparison with Ultrasonography and Clinical Joint Assessment. Clin. Radiol..

[16] da Silva Dias C., Alfieri F.M., Dos Santos A.C.A., Battistella L.R. (2021). Body Temperature and Esthesia in Individuals with Stroke. Sci. Rep..

[17] Hegedűs B. (2018). The Potential Role of Thermography in Determining the Efficacy of Stroke Rehabilitation. J. Stroke Cerebrovasc. Dis..

[18] Cabizosu A., Carboni N., Figus A., Vegara-Meseguer J.M., Casu G., Hernández Jiménez P., Martinez-Almagro Andreo A. (2019). Is Infrared Thermography (IRT) a Possible Tool for the Evaluation and Follow up of Emery-Dreifuss Muscular Dystrophy? A Preliminary Study. Med. Hypotheses.

[19] Cabizosu A., Carboni N., Martínez-Almagro Andreo A., Casu G., Ramón Sánchez C., Vegara-Meseguer J.M. (2020). Relationship between Infrared Skin Radiation and Muscular Strength Tests in Patients Affected by Emery-Dreifuss Muscular Dystrophy. Med. Hypotheses.

[20] de Freitas Zanona A., de Souza R.F., Aidar F.J., de Matos D.G., Santos K.M.B., da Conceição Paixão M., Sampaio P.Y.S., Almeida Junior H., Monte-Silva K.K. (2019). Use of Virtual Rehabilitation to Improve the Symmetry of Body Temperature, Balance, and Functionality of Patients with Stroke Sequelae. Ann. Neurosci..

[21] Johnson W., Onuma O., Owolabi M., Sachdev S. (2016). Stroke: A Global Response Is Needed. Bull. World Health Organ.

[22] (2019). GBD 2016 Neurology Collaborators Global, Regional, and National Burden of Neurological Disorders, 1990-2016: A Systematic Analysis for the Global Burden of Disease Study 2016. Lancet Neurol..

[23] Moreira D.G., Costello J.T., Brito C.J., Adamczyk J.G., Ammer K., Bach A.J.E., Costa C.M.A., Eglin C., Fernandes A.A., Fernández-Cuevas I. (2017). Thermographic Imaging in Sports and Exercise Medicine: A Delphi Study and Consensus Statement on the Measurement of Human Skin Temperature. J. Therm. Biol..

[24] Cabizosu A., Grotto D., Lopez Esteban M.J., Castañeda Vozmediano R. (2024). The assessment of neuromuscular sequels post brain damage by thermography. A pilot study. Cuest. Fisioter..

[25] Harb A., Kishner S. (2023). Modified Ashworth Scale. StatPearls.

[26] Barbachan Mansur N.S., Pereira V.F., Cunha H.C.M., Nunes C.G., Ferreira D.S., Sato V.N., Yamada A.F., Matsunaga F.T., Belloti J.C., Tamaoki M.J.S. (2021). Diagnosis of Achilles Insertional Tendinopathies by Algometry. Pain Med..

[27] Avellanal M., Riquelme I., Díaz-Regañón G. (2020). Quantitative Sensory Testing in Pain Assesment and Treatment. Brief Review and Algorithmic Management Proposal. Rev. Esp. Anestesiol. Reanim..

[28] Charlton M., Stanley S.A., Whitman Z., Wenn V., Coats T.J., Sims M., Thompson J.P. (2020). The Effect of Constitutive Pigmentation on the Measured Emissivity of Human Skin. PLoS ONE.

[29] Bouzas Marins J.C., de Andrade Fernandes A., Gomes Moreira D., Souza Silva F., Magno A., Costa C., Pimenta E.M., Sillero-Quintana M. (2014). Thermographic profile of soccer players’ lower limbs. Rev. Andal. Med. Deporte.

[30] FLIR Systems|Sistemas de Cámaras Termográficas, de Visión Nocturna e Infrarrojas|Teledyne FLIR. https://www.flir.es/.

[31] Côrte A.C., Pedrinelli A., Marttos A., Souza I.F.G., Grava J., José Hernandez A. (2019). Infrared Thermography Study as a Complementary Method of Screening and Prevention of Muscle Injuries: Pilot Study. BMJ Open Sport Exerc. Med..

[32] Carrière M.E., de Haas L.E.M., Pijpe A., Meij-de Vries A., Gardien K.L.M., van Zuijlen P.P.M., Jaspers M.E.H. (2020). Validity of Thermography for Measuring Burn Wound Healing Potential. Wound Repair. Regen..

[33] Cohen J. (2009). Statistical Power Analysis for the Behavioral Sciences.

[34] Koo T.K., Li M.Y. (2016). A Guideline of Selecting and Reporting Intraclass Correlation Coefficients for Reliability Research. J. Chiropract. Med..

[35] Kolosovas-Machuca E.S., González F.J. (2011). Distribution of Skin Temperature in Mexican Children. Skin Res. Technol..

[36] Sousa N.T.A.D., Guirro E.C.D.O., Calió J.G., Queluz M.C.D., Guirro R.R.D.J. (2017). Application of Shortwave Diathermy to Lower Limb Increases Arterial Blood Flow Velocity and Skin Temperature in Women: A Randomized Controlled Trial. Braz. J. Phys. Ther..

[37] Nowak I., Mraz M., Mraz M. (2020). Thermography Assessment of Spastic Lower Limb in Patients after Cerebral Stroke Undergoing Rehabilitation. J. Therm. Anal. Calorim..

[38] Alfieri F.M., Battistella L.R. (2018). Body Temperature of Healthy Men Evaluated by Thermography: A Study of Reproducibility. Technol. Health Care.

[39] Martínez-Noguera F.J., Cabizosu A., Marín-Pagán C., Alcaraz P.E. (2023). Body Surface Profile in Ambient and Hot Temperatures during a Rectangular Test in Race Walker Champions of the World Cup in Oman 2022. J. Therm. Biol..

[40] Ekhart D., Wicht H., Kersken T., Ackermann H., Kaczmarczyk M., Pretzsch G., Alexander H., Korf H.W. (2018). Dynamics of Core Body Temperature Cycles in Long-Term Measurements under Real Life Conditions in Women. Chronobiol. Int..

[41] Harding C., Pompei F., Bordonaro S.F., McGillicuddy D.C., Burmistrov D., Sanchez L.D. (2019). The Daily, Weekly, and Seasonal Cycles of Body Temperature Analyzed at Large Scale. Chronobiol. Int..

[42] Uematsu S., Edwin D.H., Jankel W.R., Kozikowski J., Trattner M. (1988). Quantification of Thermal Asymmetry. Part 1: Normal Values and Reproducibility. J. Neurosurg..

[43] Cabizosu A., Berenguer-Vidal R., Vegara-Meseguer J.M., Martínez-Almagro Andreo A., Maiquez Mojica V., Casu G., Carboni N. (2021). Relationship between Infrared Skin Radiation and Functional Tests in Patients Affected by Emery-Dreifuss Muscular Dystrophy: Part 2. Med. Hypotheses.

[44] Zhu C., Qiu L., Sun W., Yang C., Cong D., Wang Y., Ji G. (2023). Effect of TCM Rehabilitation Program on Activities of Daily Living in Patients with Post-Stroke Limb Spasticity: An Observational Study. Medicine.

[45] Finegan E., Li Hi Shing S., Siah W.F., Chipika R.H., Chang K.M., McKenna M.C., Doherty M.A., Hengeveld J.C., Vajda A., Donaghy C. (2020). Evolving Diagnostic Criteria in Primary Lateral Sclerosis: The Clinical and Radiological Basis of “Probable PLS”. J. Neurol. Sci..

[46] Kim M.S., Seo D.H., Lim M.H., Kim T.U., Lee S.J., Hyun J.K. (2012). Skin Temperature Changes Following Sciatic Nerve Injury in Rats. J. Neurotrauma.

[47] Ra J.Y., An S., Lee G.-H., Kim T.U., Lee S.J., Hyun J.K. (2013). Skin Temperature Changes in Patients with Unilateral Lumbosacral Radiculopathy. Ann. Rehabil. Med..

[48] Wagatsuma A., Osawa T. (2006). Time Course of Changes in Angiogenesis-Related Factors in Denervated Muscle. Acta Physiol..

[49] Whitney D.G., Singh H., Miller F., Barbe M.F., Slade J.M., Pohlig R.T., Modlesky C.M. (2017). Cortical Bone Deficit and Fat Infiltration of Bone Marrow and Skeletal Muscle in Ambulatory Children with Mild Spastic Cerebral Palsy. Bone.

[50] Gomes G.G.C., Palinkas M., da Silva G.P., Gonçalves C.R., Lopes R.F.T., Verri E.D., Fabrin S.C.V., Fioco E.M., Siéssere S., Regalo S.C.H. (2022). Bite Force, Thickness, and Thermographic Patterns of Masticatory Muscles Post-Hemorrhagic Stroke. J. Stroke Cerebrovasc. Dis..

[51] Candelario-Jalil E., Dijkhuizen R.M., Magnus T. (2022). Neuroinflammation, Stroke, Blood-Brain Barrier Dysfunction, and Imaging Modalities. Stroke.

[52] Karaszewski B., Carpenter T.K., Thomas R.G.R., Armitage P.A., Lymer G.K.S., Marshall I., Dennis M.S., Wardlaw J.M. (2013). Relationships between Brain and Body Temperature, Clinical and Imaging Outcomes after Ischemic Stroke. J. Cereb. Blood Flow Metab..

[53] Naito E., Nakashima T., Kito T., Aramaki Y., Okada T., Sadato N. (2007). Human Limb-Specific and Non-Limb-Specific Brain Representations during Kinesthetic Illusory Movements of the Upper and Lower Extremities. Eur. J. Neurosci..

[54] Pontén E.M., Stål P.S. (2007). Decreased Capillarization and a Shift to Fast Myosin Heavy Chain IIx in the Biceps Brachii Muscle from Young Adults with Spastic Paresis. J. Neurol. Sci..

[55] Salom-Moreno J., Sánchez-Mila Z., Ortega-Santiago R., Palacios-Ceña M., Truyol-Domínguez S., Fernández-de-las-Peñas C. (2014). Changes in Spasticity, Widespread Pressure Pain Sensitivity, and Baropodometry after the Application of Dry Needling in Patients Who Have Had a Stroke: A Randomized Controlled Trial. J. Manip. Physiol. Ther..

[56] Dibai Filho A.V., de Oliveira A.K., Oliveira M.P., Bevilaqua-Grossi D., de Jesus Guirro R.R. (2021). Relationship between Pressure and Thermal Pain Threshold, Pain Intensity, Catastrophizing, Disability, and Skin Temperature over Myofascial Trigger Point in Individuals with Neck Pain. Rev. Assoc. Med. Bras..

[57] Alfieri F.M., Lima A.R.S., Battistella L.R., de Oliveira Vargas e Silva N.C. (2019). Superficial Temperature and Pain Tolerance in Patients with Chronic Low Back Pain. J. Bodyw. Mov. Ther..

[58] Haddad D.S., Brioschi M.L., Arita E.S. (2012). Thermographic and Clinical Correlation of Myofascial Trigger Points in the Masticatory Muscles. Dentomaxillofac. Radiol..

[59] Sempere-Rubio N., Aguilar-Rodríguez M., Inglés M., Izquierdo-Alventosa R., Serra-Añó P. (2021). Thermal Imaging Ruled out as a Supplementary Assessment in Patients with Fibromyalgia: A Cross-Sectional Study. PLoS ONE.

[60] Zhang Y.-H., Xu H.-R., Wang Y.-C., Hu G.-W., Ding X.-Q., Shen X.-H., Yang H., Rong J.-F., Wang X.-Q. (2022). Pressure Pain Threshold and Somatosensory Abnormalities in Different Ages and Functional Conditions of Post-Stroke Elderly. BMC Geriatrics.

